# Supplementary material for: Aberrant interaction of FUS with the U1 snRNA provides a molecular mechanism of FUS induced amyotrophic lateral sclerosis
Source: Nat Commun. 2020 Dec 11;11:6341. doi: 10.1038/s41467-020-20191-3 (PMC7733473; doi:10.1038/s41467-020-20191-3)
Supplement: Supplementary file 1 — Supplementary Information [file 41467_2020_20191_MOESM1_ESM.pdf]

Supplementary information for

Aberrant interaction of FUS with the U1 snRNA provides a molecular mechanism of FUS induced Amyotrophic Lateral Sclerosis

Jutzi, Campagne, et al

Supplementary Figures

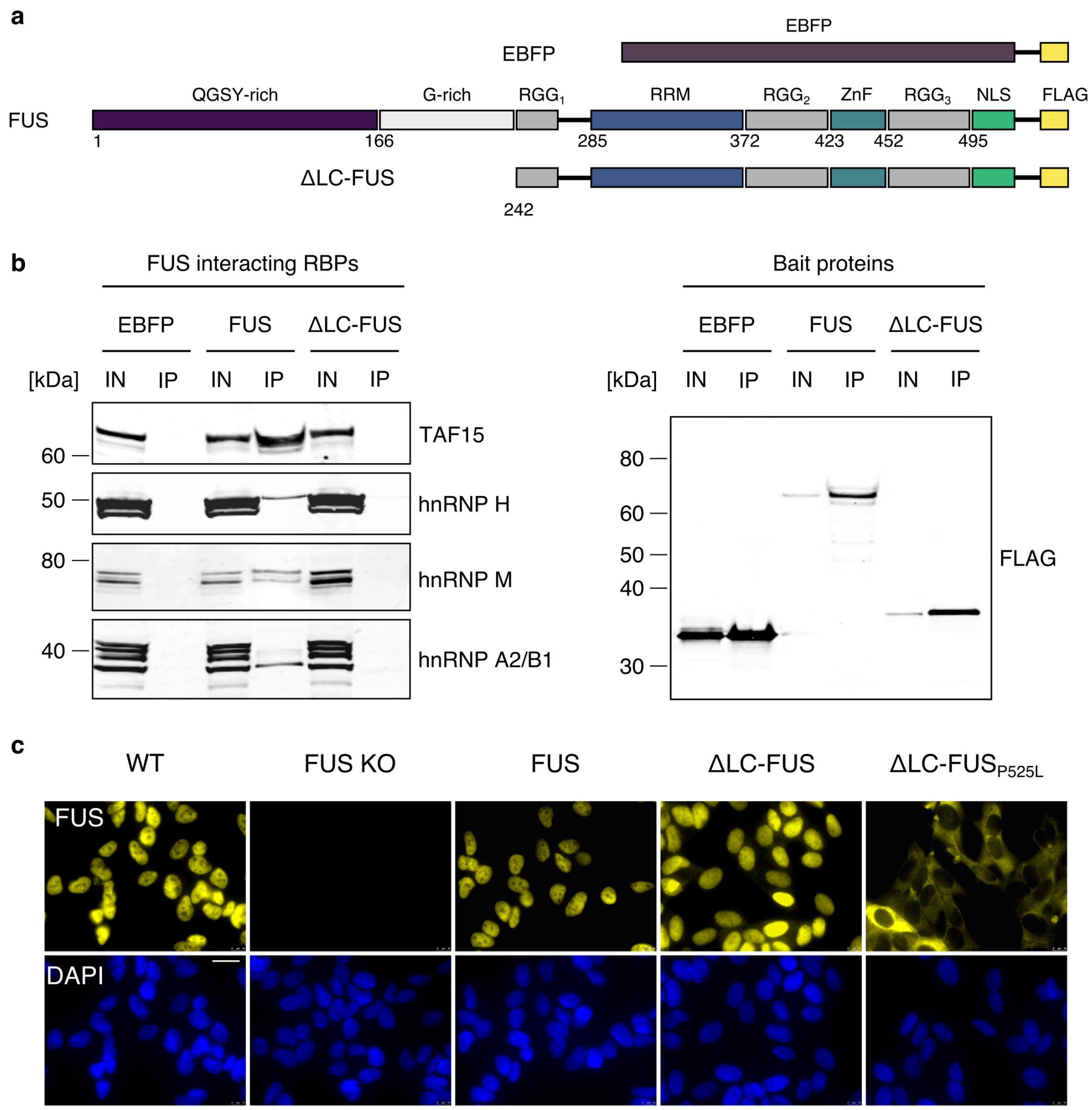

Supplementary Figure 1 I An RBD-centric FUS CLIP approach.

(**a**) Schematic representation of the FLAG-tagged constructs used for IP. (**b**) FLAG-tagged FUS constructs or EBFP (negative control) were precipitated from HeLa FUS KO cells, and purified complexes were separated by SDS-Page followed by analysis by western blotting using the respective antibodies. Input corresponding to 10^5 cell equivalents was loaded, IP fractions correspond to 5 x 10^6 cell equivalents. n = 1 (**c**) Uncropped images of immunofluorescence staining of FUS in the SH-SY5Y cell lines used for CLIP, corresponding to Fig. 1d-f, including a WT and FUS knockout control. FUS was detected using anti-FUS antibodies. Nuclei were counterstained with DAPI. Scale bar = 10μm, n = 1.

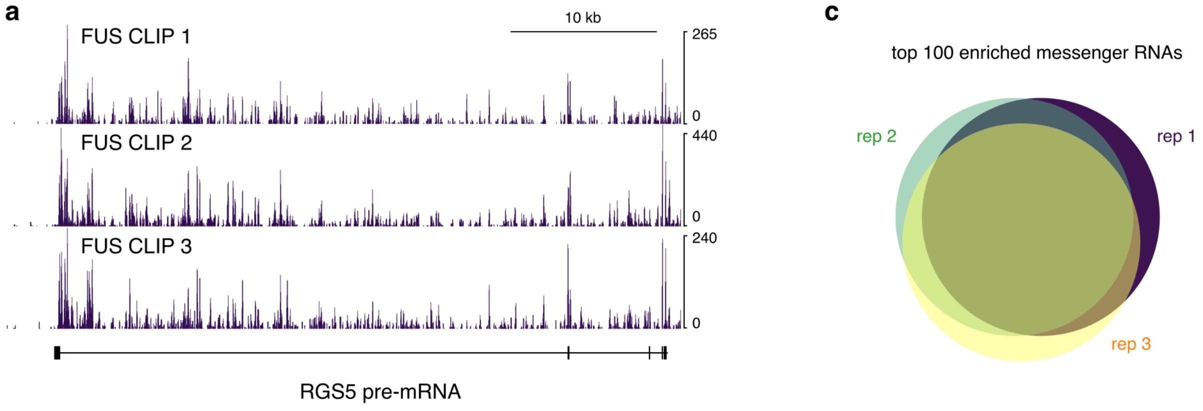

b

FUS CLIP rep1

X / Y	A	C	G	N	T
A		7.831%	2.534%	0.01%	2.619%
C	2.867%		1.147%	0.007%	4.845%
G	5.164%	2.637%		0.009%	5.848%
N	0%	0%	0%		0%
T	4.611%	8.927%	3.136%	0.011%	
INS	1.653%	3.75%	1.613%	0.001%	1.683%
DEL	7.658%	3.701%	5.626%	0%	22.112%

FUS CLIP rep2

X / Y	A	C	G	N	T
A		7.69%	2.522%	0.012%	2.478%
C	3.285%		1.015%	0.009%	4.774%
G	5.592%	2.492%		0.01%	5.606%
N	0%	0%	0%		0%
T	4.892%	8.551%	2.932%	0.012%	
INS	1.487%	2.532%	1.307%	0.001%	1.36%
DEL	8.205%	4.043%	6.161%	0.001%	23.032%

FUS CLIP rep3

X / Y	A	C	G	N	T
A		8.09%	2.497%	0.01%	2.54%
C	2.926%		1.022%	0.007%	4.779%
G	5.226%	2.528%		0.008%	5.593%
N	0%	0%	0%		0%
T	4.645%	9.135%	2.944%	0.01%	
INS	1.52%	3.601%	1.273%	0.001%	1.449%
DEL	7.935%	3.833%	5.863%	0.001%	22.566%

Supplementary Figure 2 l CLIP-Seq reveals U1 snRNA as a major FUS target.

(**a**) Genome browser view showing CLIP read distribution on the RGS5 pre-mRNA for three biological replicates. (**b**) Density plots showing mutation frequencies in CLIP reads for three biological replicates [1]. (**c**) Proportional Venn diagram showing overlap of top 100 enriched transcripts between replicates.

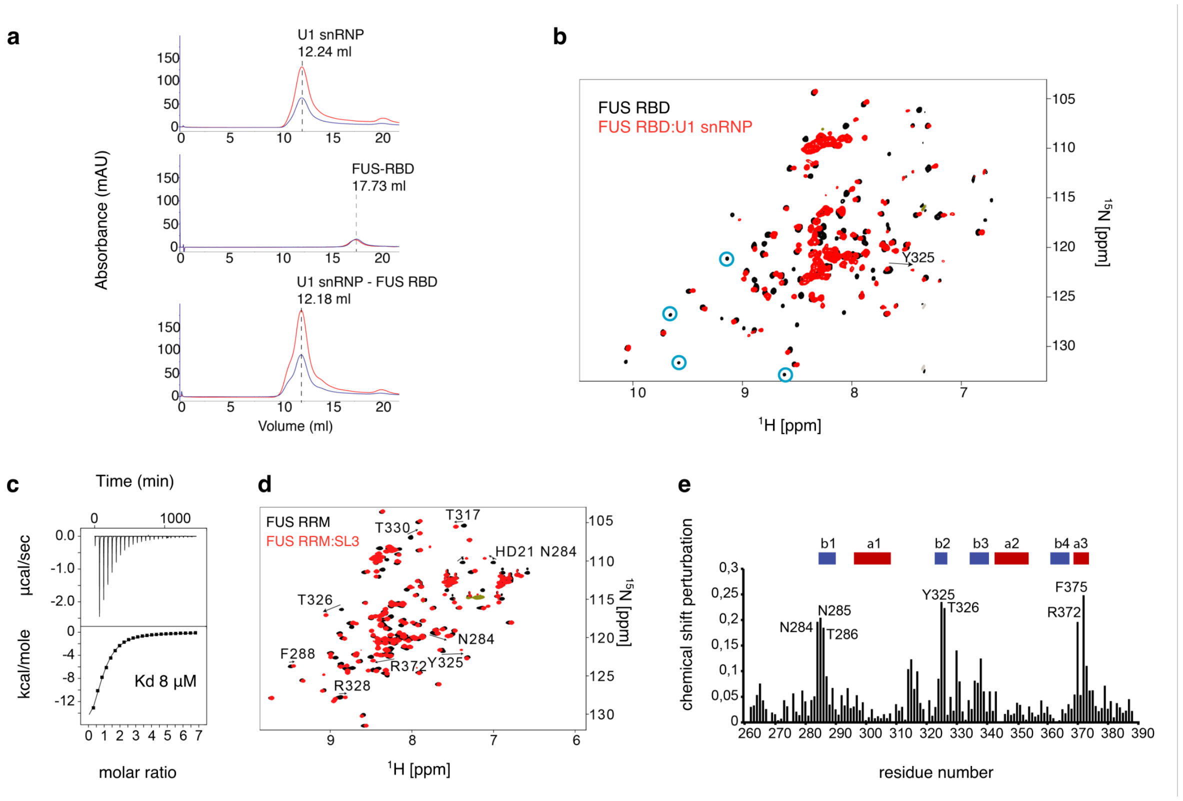

Supplementary Figure 3 I *In vitro* characterisation of the FUS – U1 snRNP interaction.

(**a**) FUS-RBD co-elutes with U1 snRNP in analytical size exclusion chromatography. For each chromatogram, absorbance at 280 and 260 nm are shown in blue and red, respectively. (**b**) Overlay of the 2D 1H-15N TROSY-HSQC spectra of ^{15}N, ^{2}H FUS-RDB free (black) or bound to U1 snRNP (red). The blue circles indicate resonances of the zinc finger domain that broaden upon addition of U1 snRNP. n = 3. (**c**) ITC measurements of FUS-RRM with SL3. n = 3. (**d**) Overlay of the ^{2}D ^{1}H-^{15}N HSQC spectra of FUS-RRM free (black) or in complex with SL3 (red). (**e**) Bar plot showing the combined chemical shift changes of FUS-RRM amide groups as a function of the residue number.

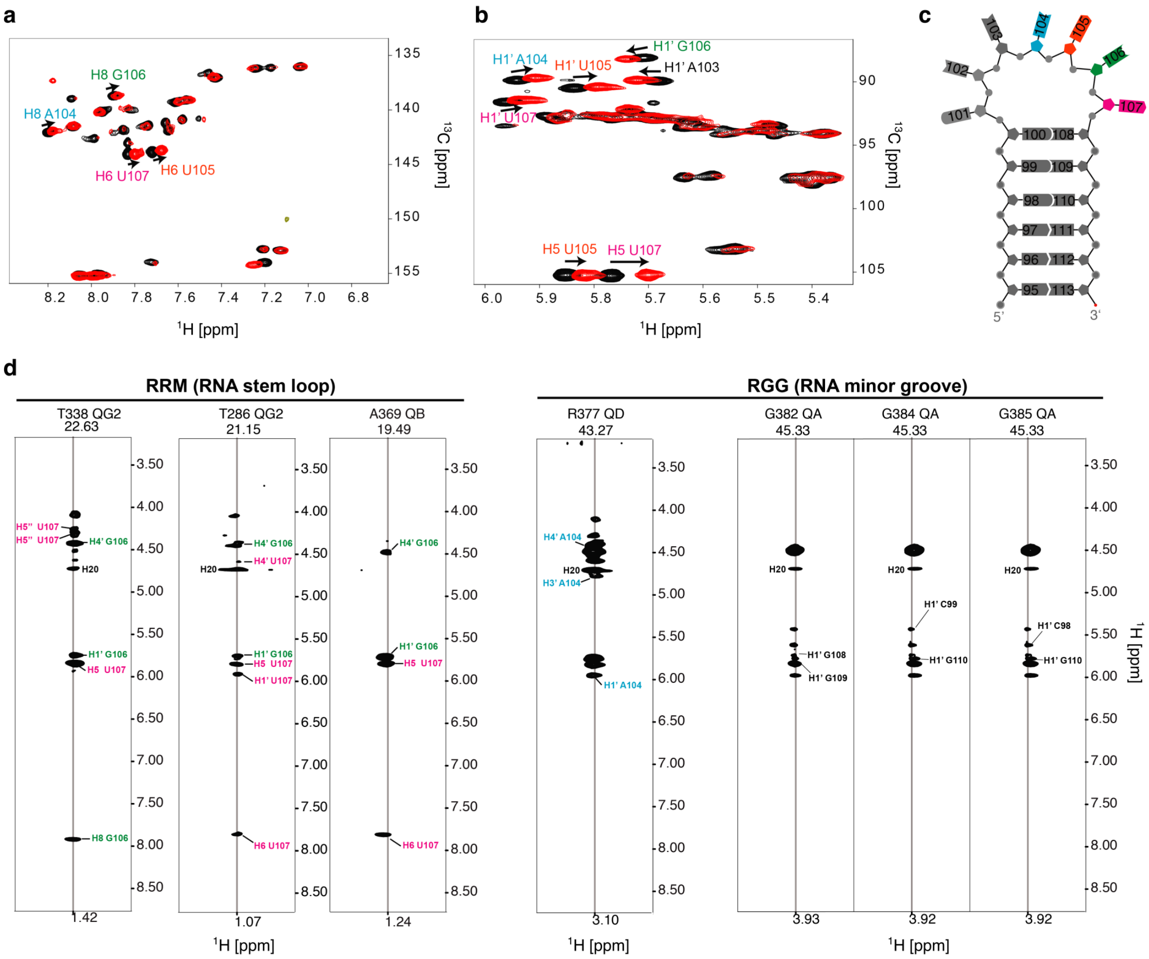

Supplementary Figure 4 I Structural basis of the FUS - U1 snRNP interaction.

(**a**) Overlay of the 2D ^{1}H ^{13}Caro HSQC spectra of SL3 before (black) and after addition of FUS-RRM (red). (**b**) Overlay of the 2D ^{1}H ^{13}Cali HSQC spectra of SL3 before (black) and after addition of FUS-RRM (red). (**c**) Schematic representation of SL3. (**d**) Strips extracted from the 3D ^{13}C-(F1-edited, F3-filtered) NOESY HSQC showing intermolecular NOEs coming from the RRM and the RRG2 part of the protein.

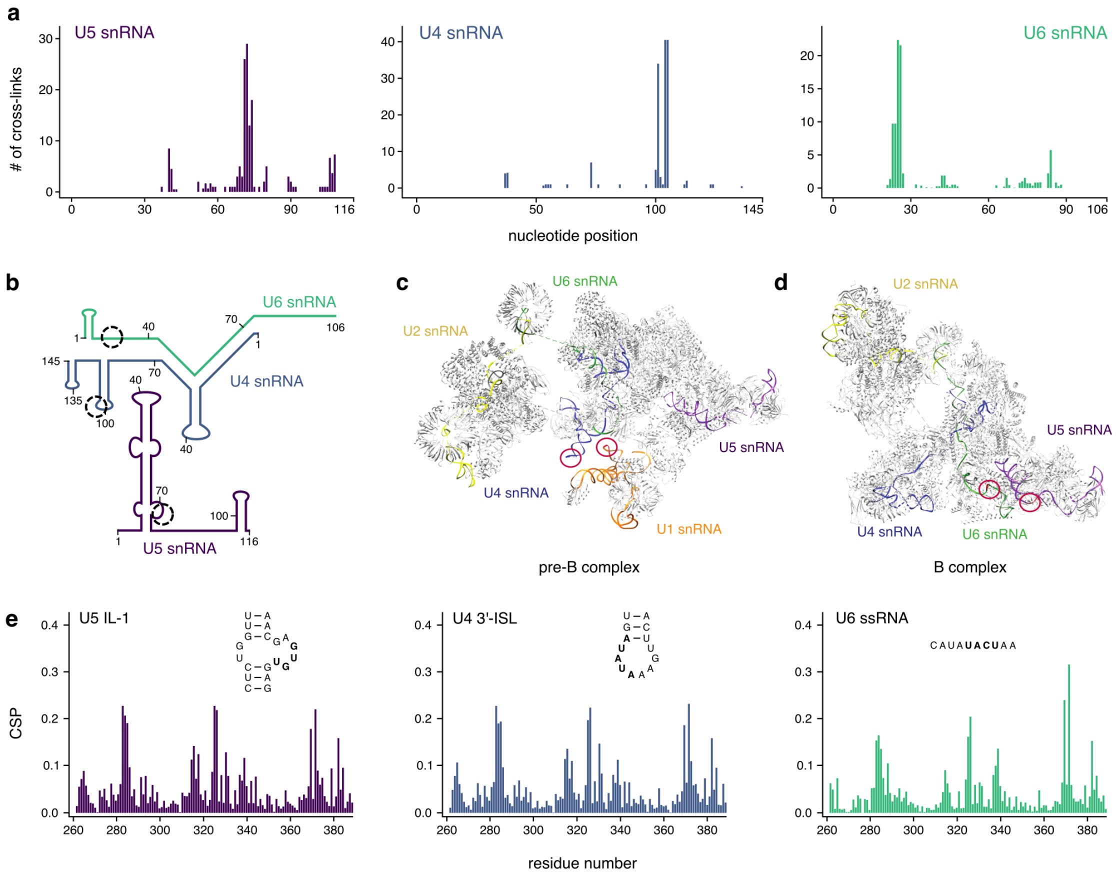

Supplementary Figure 5 I FUS directly interacts with the U4, U5 and U6 snRNAs.

(**a**) Bar plots displaying the number of crosslinks along the primary sequences of the U5, U4 and U6 snRNAs. (**b**) Schematic representation of U4, U5 and U6 snRNAs. (**c**) ribbon representation of the pre-B spliceosome complex [2]. The CLIP induced deletion sites on U1 (orange) and U4 snRNA (blue) are indicated by red circles. (**d**) Ribbon representation of the B spliceosome complex [3]. The CLIP induced deletion sites on U5 (magenta) and U6 snRNA (green) are indicated by red circles. (**e**) Bar plots displaying chemical shift differences in FUS-RRM upon incubation with RNA fragments of the U4, U5 and U6 snRNA. n = 2.

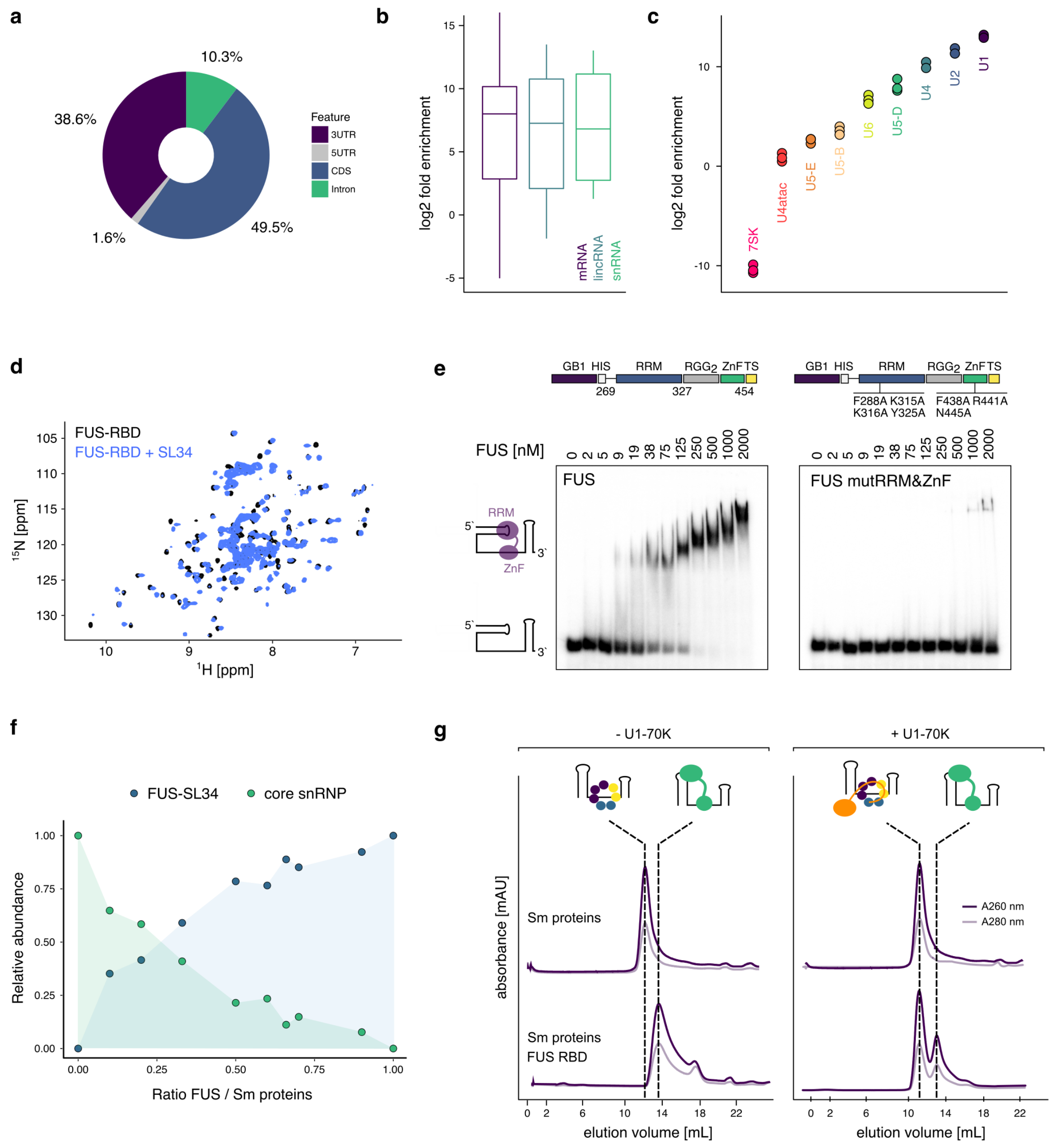

Supplementary Figure 6 I The FUS-U1 snRNA interaction is altered in the cytoplasm.

(**a**) Read distribution of ΔLC-FUS$_{P525L}$ CLIP experiment according to annotated transcript region. (**b**) Box plot showing the distribution of enrichments values separated by biotype. Plot displays median lines, interquartile range (IQR) boxes and 1.5 x IQR whiskers. n = 1,337 (lincRNA), 103,556 (mRNA), 96 (snRNA), examined in three biological replicates. (**c**) Dot plot showing mean enrichment values of binding sites located in individual snRNAs for three biological replicates. (**d**) Overlay of the ^{1}H-^{15}N HSQC spectra of FUS-RBD (aa 269-454) in the absence (black) or presence (blue) of SL34 RNA. n

= 3 (**e**) Electrophoretic mobility shift assays were performed with WT or RNA-binding deficient FUS (aa 269-454) and ^{32}P-labelled SL34 RNA. n = 2 (**f**) Quantification of core snRNP assembly assay in Fig. 4e. n = 3 (**g**) Core snRNPs were assembled *in vitro* with and without FUS-RBD and U1-70K (aa1-216). The resulting complexes were separated by analytical size exclusion chromatography. Absorbance at 260 and 280 nm is shown. n = 2.

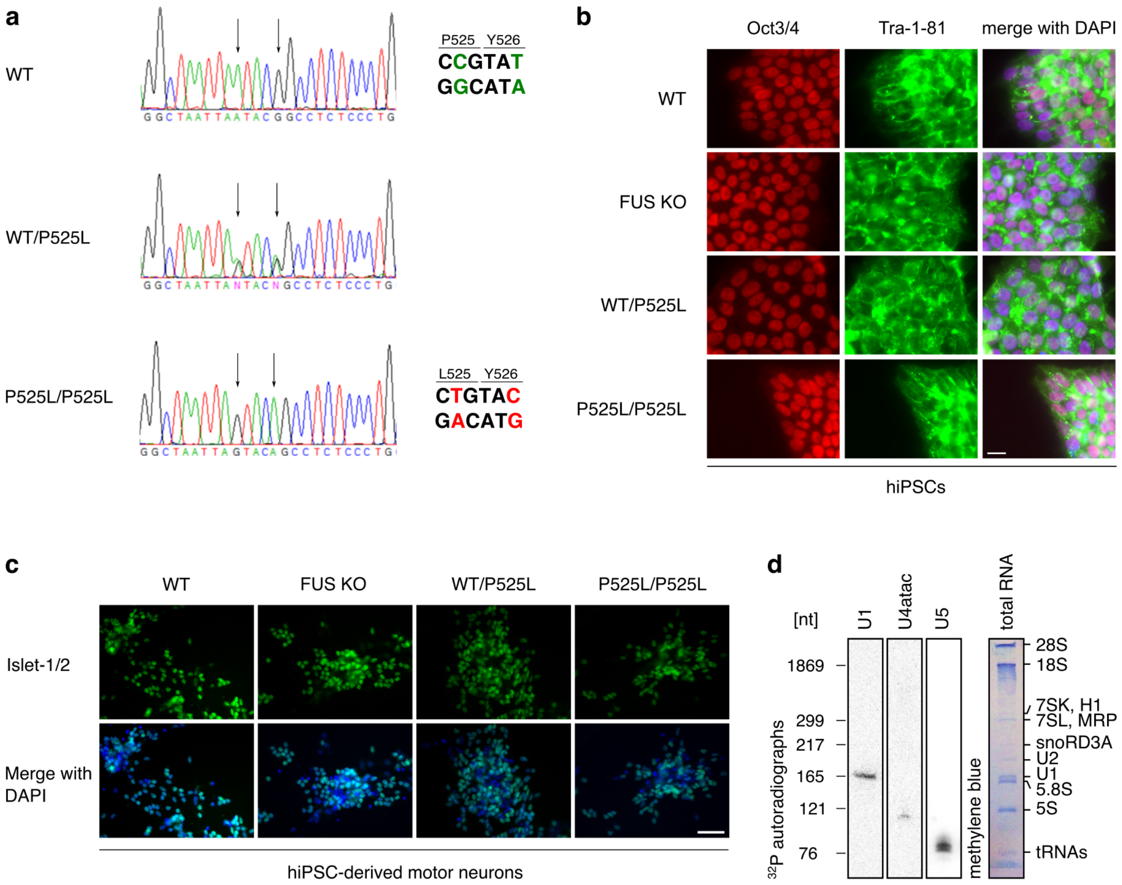

Supplementary Figure 7 l Characterisation of genome-edited hiPSC lines.

(**a**) Sequencing chromatograms of WT, heterozygous and homozygous FUS P525L hiPSCs confirming the presence of the desired mutations. Arrows indicate the nucleotide positions that were edited. (**b**) Immunostaining visualising expression of the stem cell markers Oct3/4 and Tra-1-81 using antibodies. Scale bar = 20 μm, n = 1. (**c**) Immunostaining showing expression of the motor neuron marker Islet-1/2 (green). Nuclei were counterstained with DAPI. Scale bar = 50 μm, n = 1. (**d**) Autoradiographs showing specificity of ^{32}P-labelled anti-sense probes under the hybridisation conditions used for FISH. Total RNA was separated by denaturing Urea-PAGE, blotted onto a nylon membrane. RNA was visualised using methylene blue. n = 1.

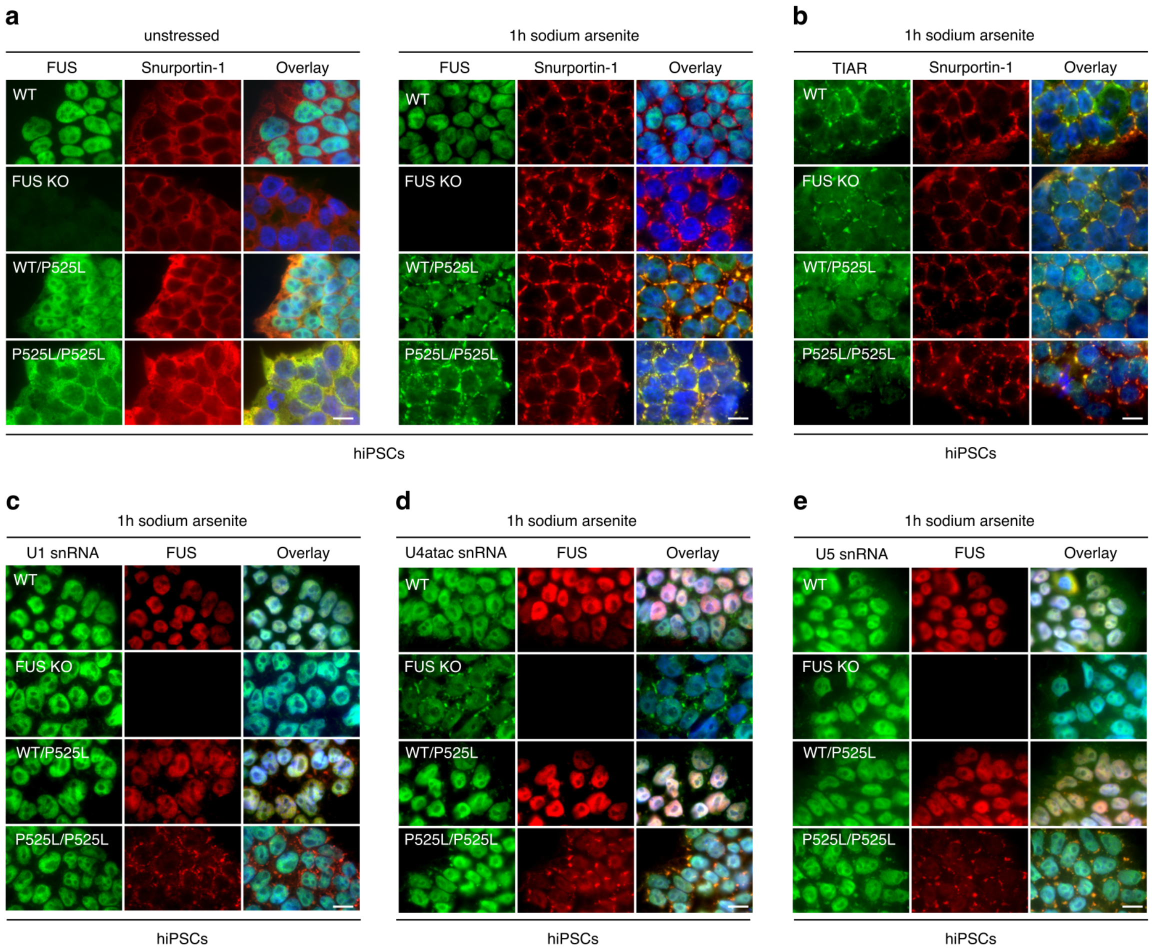

Supplementary Figure 8 I Core snRNPs localise to stress granules upon oxidative stress. (**a**) Immunostainings in hiPSCs with and without sodium arsenite treatment. FUS (green) and Snurportin-1 (red) were visualised using respective antibodies. Nuclei were counterstained with DAPI. Scale bar = 15 μm, n = 2. (**b**) Immunostaining in hiPSCs showing localisation of TIAR (green) and Snurportin-1 (red) upon stress. Scale bar = 15 μm, n = 1 (**c**) Combined FISH and immunostaining in hiPSCs following arsenite treatment. U1 snRNA (green) was visualised using labelled anti-sense probes respective antibodies. FUS (red) was detected using antibodies. Nuclei were counterstained with DAPI. Scale bar = 15 μm, n = 1 (**d**) Combined FISH and immunostaining visualising FUS (red) and U4atac snRNA (green) in arsenite-challenged hiPSCs. Scale bar = 15 μm, n = 1 (**e**) Combined FISH and immunostaining visualising FUS (red) and U5 snRNA (green) in arsenite-challenged hiPSCs. Scale bar = 15 μm, n = 1.

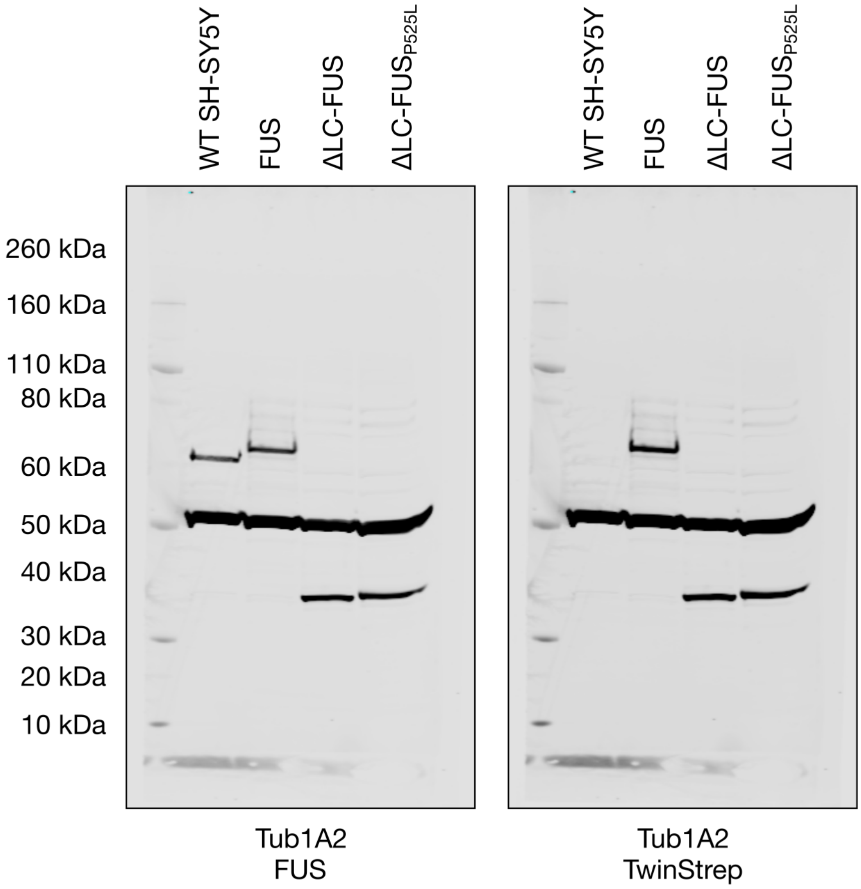

Supplementary Figure 9 l Uncropped western blots.

Full scan images of the western blots shown in figure 1c.

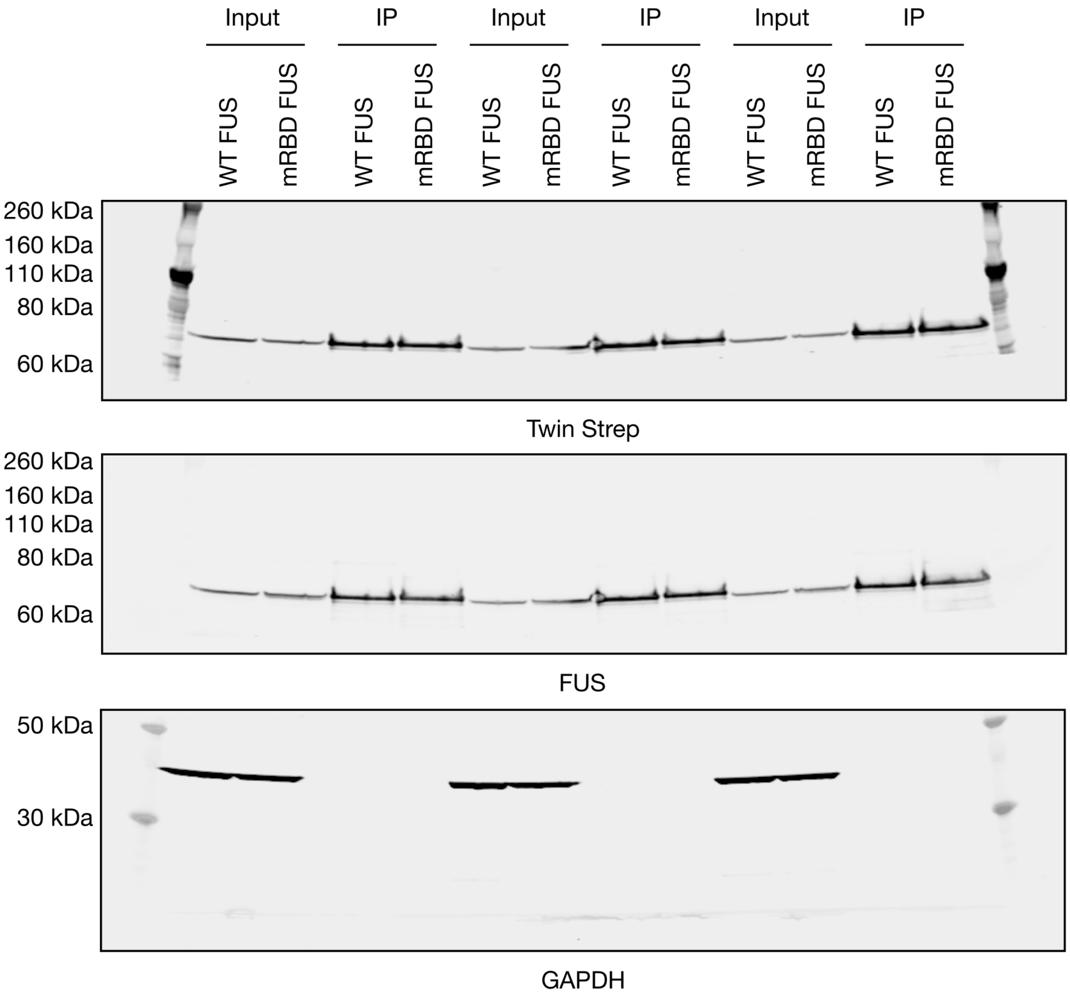

Supplementary Figure 9 (continued) I Uncropped western blots.

Full scan images of the western blots shown in figure 2g.

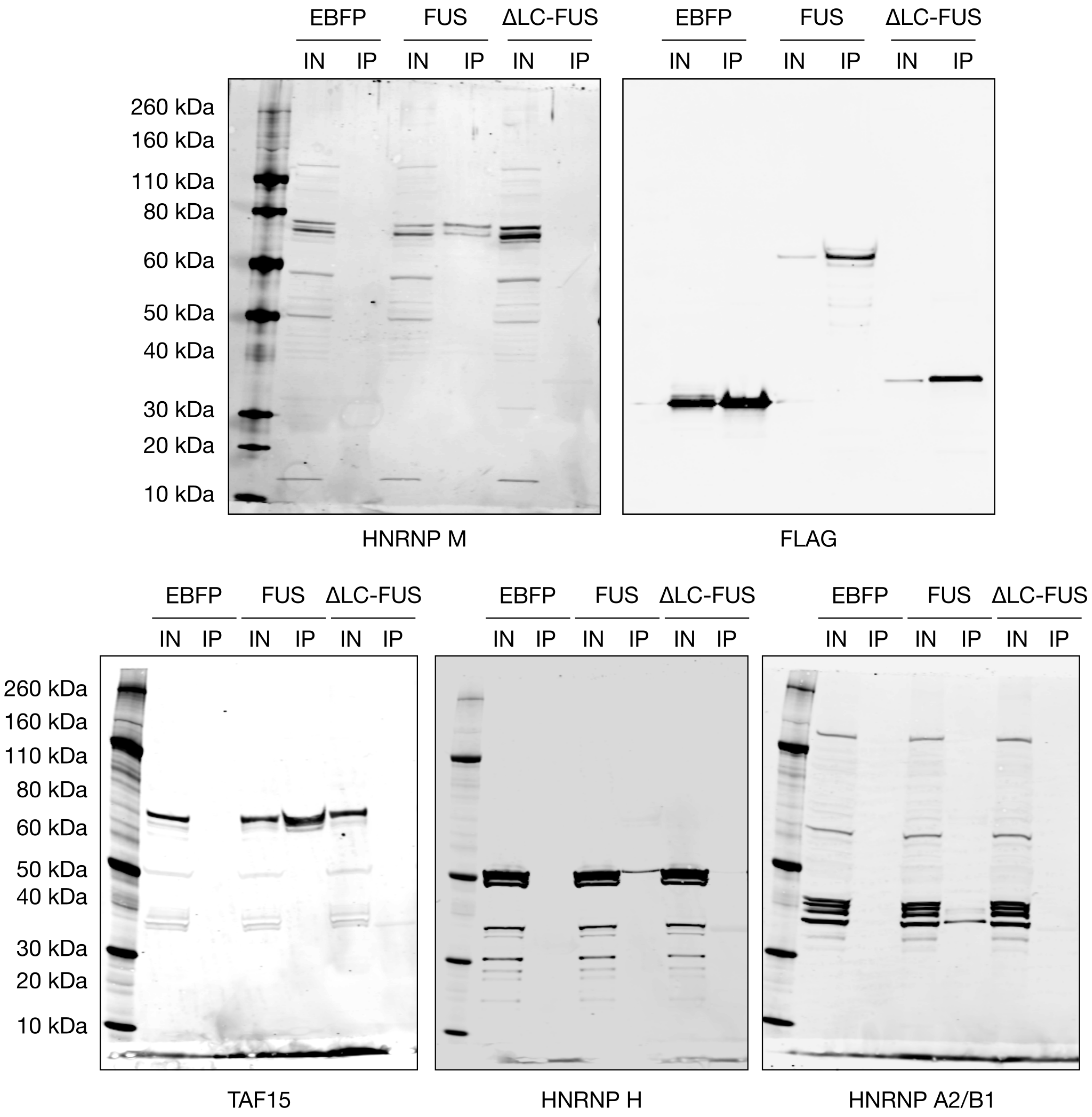

Supplementary Figure 9 (continued) I Uncropped western blots.

Full scan images of the western blots shown in supplementary figure 1b.

Supplementary Tables

Supplementary Table 1 I Oligonucleotides used in this study.

Oligo ID	Oligo Name	Use	Sequence
sre10	FUS P525 locus fwd	Sequencing	GTGGCTCTCACATGGGTAAG
sre11	FUS P525 locus rev	Sequencing	AAAGACCCAGAGTGGCTAAG
sre125	FUS P525 seq	Sequencing	GGGTGATCAGGAATTGGAAGG
mdr738	P525oligo fwd	Cloning	CACCGGGAGCCAGGCTAATTAATA
mdr739	P525oligo rev	Cloning	AAACTATTAATTAGCCTGGCTCCC
mdr732	P525L target f	Cloning	TCGCAGGGAGAGGCCGTATTAATTAGC CTGGCTCCCCAGGTTCTGGAAACGT
mdr733	P525L target r	Cloning	TTCCAGAACCTGGGGAGCCAGGCTAAT TAATACGGCCTCTCCCTGCGAAGCT
	Universal RT primer	RT	GCTGTCAACGATACGCTACGTAACGG CATGACAGTGTTTTTTTTTTTTTTTTTVN
	Universal reverse primer	RT-qPCR	GCTGTCAACGATACGCTACGTAAC
mdr597	U1 snRNA qPCR f	RT-qPCR	TTCGCGCTTTCCCCTGAA
mdr591	U11 snRNA qPCR f	RT-qPCR	GTCCCGGCGCCCTTTAA
mdr247	7SL RNA qPCR f	RT-qPCR	CCTGGGCAACATAGCGAGAC
dj215	FUS P525L rev NES	cloning	TTTTGGATCCGATGTCCAGGCCGGCCAGCTTCAGGG CCAGGTAAAGCCGTTCCCGTCTGTCCTGCCGGTGCTC
mdr710	Eco_Xb fwd FUSop	cloning	AAAAGAATTCTCTAGAGCCGCCACCATGAGAGGAAGAG
mdr749	Opt FUS Eco F	cloning	AATGAATTCGCCGCCACCATGGCCAGCAACGACTACACC
mdr750	OptFUS rev Bam	cloning	AATGGATCCTCATCAGTAAGGCCGTTCCC
mdr761	qc mat g958a	mutagenesis	GGAGAGGCTGTACTAATTAGCCTGGCTCCCC
mdr762	qc mat cc983_4	mutagenesis	GGCTCCCCAGGTTCTGGAACAGCTTTTTGTCCTGTA
mdr763	qc mat cg937_40	mutagenesis	GTGAGCACAGACAGGATCGCAGGGAGAGGCTG
mdr71	QC FUS RRM YtoA	mutagenesis	GGACAGCCCATGATTAATTTGGCCACAGACAGGGAAACTG
mdr740	qc FUS F288A	mutagenesis	CAGACAACAACACCATCGCTGTGCAAGGCCTGGGTG
mdr741	qc FUS F438-N445	mutagenesis	GTTTAGGGGCCTTACACTGGGCGCATTCATTCGCCCAAGA GGCGTTCATATTCTCAC
mdr742	QC RRM KK loop	mutagenesis	GCCAAATTAATCATGGGCTGTCCCGTTGCTGCGTTTGTCTT AATAATACCAATCTGCTTGAAGTAATCA

Supplementary Table 2 l Antibodies used in this study.

Antibody	Species	Source	Use	Dilution
FUS (4H11)	Mouse	Santa Cruz, sc-4711	WB / IF	1:500 / 1:200
FUS	Rabbit	Novus Biologicals, NB100-565	IF	1:500
FUS (serum 6)	Rabbit		WB	1:7500
Snurportin-1 (B-12)	Mouse	Santa Cruz, sc-137133	IF	1:500
TIAR (C-18)	goat	Santa Cruz, sc-1749	IF	1:200
Islet-1/2	mouse	DSHB, 39.4D5	IF	1:100
β-Tubulin III (2G10)	Mouse	Sigma, T8578	IF	1:500
TwinStrep	Mouse	IBA life sciences, 2-1507-001	WB	1:2000
GAPDH	Mouse	Santa Cruz, sc-32233	WB	1:500
Tub1A2	Mouse	Sigma, T9028	WB	1:5000
hnRNP H	Rabbit	Bethyl Laboratories, A300-511A	WB	1:5000
hnRNP A2/B1	Rabbit	Abcam, ab31645	WB	1:500
hnRNP M	Rabbit	Proteintech, 26897-1-AP	WB	1:500
TAF15	Rabbit	Abcam, ab133760	WB	1:1000
FLAG (M2)	Mouse	Sigma, F1804	WB	1:1000
Mouse 680LT	Goat	LI-COR, 926-68020	WB	1:10000
Mouse 800CW	Goat	LI-COR, 926-32210	WB	1:10000
Rabbit 680LT	Goat	LI-COR, 926-68021	WB	1:10000
Rabbit 800CW	Goat	LI-COR, 926-32211	WB	1:10000
Mouse AF488	Donkey	Thermo Fisher, A21202	IF	1:1000
Mouse AF546	Donkey	Thermo Fisher, A10036	IF	1:1000
Rabbit AF488	Donkey	Thermo Fisher, A21206	IF	1:1000
Rabbit AF546	Donkey	Thermo Fisher, A10040	IF	1:1000
Mouse AF488	Chicken	Thermo Fisher, A-21200	IF	1:1000

Supplementary Methods

Plasmids

To create the pLVX-optFUS$_{242-526}$-GSG$_{15}$-TS vector, the optimised coding sequence of FUS amino acids 242-526 followed by a GSG15 linker and Twin-Strep tag (WSHPQFEK-GGGSGGGSGGS-WSHPQFEK) was ordered as gene synthesis [GeneArt, Life Technologies] and cloned into the EcoR1, Not1 sites of pLVX-IRES-Puro [Clonetech]. The pLVX-optFUS-GSG$_{15}$-TS vector was generated by cloning the optimised FUS amino acids 1-323 coding sequence from pLVX-optFUS-UT into pLVX-optFUS$_{242-526}$-GSG$_{15}$-TS using EcoR1, BsrG1 sites. pLVX-optFUS-UT was generated by amplifying the optimised FUS coding sequence from pLVX-TS-optFUS using the primers mdr749 and mdr750 and cloned into the EcoR1, BamH1 sites of pLVX-IRES-Puro. pLVX-TS-optFUS was generated by introducing a string encoding the Twin-Strep-tagged n-terminal (aa 1-150) optimised coding sequence of FUS [GeneArt, Life Technologies] into pLVX-optFUS-deltaFET using the EcoR1, PshA1 sites. pLVX-optFUS-deltaFET was generated by transfer of the optimized FUS coding sequence lacking amino acids 36-66 from pMS-RQ-optFUS-deltaFET [GeneArt, Life Technologies] to pLVX-IRES-Puro using Xba1/BamH1 sites. The pLVX-optFUS$_{242-526}$-P525L-NES-GSG$_{15}$-TS plasmid was produced as follows: The FUS$_{242-526}$ coding sequence was amplified from pLVX-optFUS$_{242-526}$-GSG$_{15}$-TS using the primers mdr710 and dj215 and inserted into the EcoR1, BamH1 sites of pLVX-optFUS$_{242-526}$-RGGtoKGG-GSG$_{15}$-TS.

pLVX-optFUS$_{242-526}$-RGGtoKGG-GSG$_{15}$-TS was generated by transfer of the coding sequence from pUC57-optFUS$_{242-526}$-RGGtoKGG-GSG$_{15}$-TS (digested with Xba1, Bgl2) [General Biosystems] to pLVX-IRES-Puro using its Xba1, BamH1 sites. pLVX-TS-FUS-mutRBD was generated by quick change mutagenesis from pLVX-TS-FUS using the primers mdr71, mdr740, mdr741 and mdr742. pcDNA3.1-EBFP-GSG15-FLAG and pcDNA3.1-FUS-GSG15-FLAG were described in [4]. pcDNA3.1-FUS$_{242-526}$-GSG$_{15}$-FLAG was ordered as gene synthesis [GeneArt, Life Technologies]. The U1 snRNA sequence was ordered as string [GeneArt, Life Technologies] and inserted into the BamH1, Hind3 sites of pcDNA3.1. pUC57-U4atac and pUC57-U5-E were ordered as gene synthesis [General Biosystems]. The plasmids for the expression of GB1-tagged FUS constructs (pET24A-optFUS612-

WT and pET24A-optFUS612-mutRBD) were ordered as gene synthesis [General Biosystems]. The other plasmids allowing the bacterial expression of recombinant FUS constructs (pET28a-FUS RRM-RGG2 and pET28a-FUS-RGG2-ZnF) as well as the purification procedures have been already described [5]. Plasmids, expression and purification protocols for the U1 snRNP protein components were also already described [6]. pUC19-HH-U1snRNA were ordered as gene synthesis [General Biosystems], it contains an insert encoding for the T7 promoter, a hammerhead ribozyme in 5' and the sequence of U1 snRNA flanked by a Sal1 restriction site. pUC19-U1 SL34 was generated by cloning a dsDNA fragment encoding for the T7 promoter followed by the sequence of SL34. pUC19-U1 SL34mut was generated by quick change mutagenesis by using as template the pUC19-U1SL34. pCRISPREF1a-SpCas9 was described in [7]. To generate pCRISPREF1a-SpCas9 P525 two complementary oligonucleotides (mdr738, mdr739) were subsequently phosphorylated using T4 PNK, annealed by heating to 95 °C and cooling to room temperature creating 5' overhangs on both ends which were used to ligate into the BbsI sites of pCRISPREF1a-SpCas9. The donor plasmid for the P525L gene editing was created as follows: The endogenous FUS sequence encompassing 488 bp upstream and 563 bp downstream of the guide targeting sequence containing the P525L mutation and several silent mutations used for TALENs was ordered by gene synthesis in pUC57 [Cellectis]. To serve as a template for CRISPR-mediated editings, these silent mutations were removed by quick change mutagenesis using the mdr761, mdr762 and mdr763 primers. The pRR-Puro P525 plasmid was cloned by inserting oligonucleotides mdr732, mdr733 into the Sac1, Aat2 sites of pRR-Puro. The pRR-Puro plasmid is described in [8].

RNA preparation and *in vitro* U1 snRNP reconstitution

The U1 snRNA, U1SL34 and U1SL34mut were transcribed from their respective linearised plasmid templates (cleaved by SalI for U1snRNA or by BsaI for U1SL34 and U1SL34mut) using in house produced T7 run off. The transcription mixture was then loaded into an HPLC system and purified as previously described [5]. The *in vitro* reconstitution of U1 snRNP was performed as already described [9]. In brief, purified Sm proteins were spontaneously assembled on the *in vitro* transcribed U1 snRNA. The Sm core was then stabilized by the addition of recombinant U1-70K (1-216). The

pre-particles were further decorated by adding full-length U1-A and U1-C. Finally, the reconstituted U1 snRNPs were purified by size exclusion chromatography. The RNA fragments of U4 snRNA and U5 snRNA were produced by *in vitro* transcription using as template the following oligonucleotides:

non-template strand for U4 snRNA:

5'-TCTAATACGACTCACTATAGGACTTGAAATATAGTCC-3'

template strand for U4 snRNA:

5'-GGACTATATTTCAAGTCCTATAGTGAGTCGTATTAGA-3'

non-template strand for U5 snRNA:

5'-TCTAATACGACTCACTATAGGCTCTGGTTTCTTCGGGAACAACTCTGAGCC-3'

template strand for U5 snRNA:

5'-GGCTCAGAGTTGTTCCCGAAGAAACCAGAGCCTATAGTGAGTCGTATTAGA-3'.

The U5 snRNA target being an internal bulge, the two segments of U5 snRNA 4-18 and 59-77 were linked by a UUCG tetraloop to obtain the correct tertiary structure. The U6 snRNA fragment containing the nucleotides 21-28 was chemically synthetized by Dharmacon.

Production of isotope labelled proteins for NMR spectroscopy

For uniform isotope labelling (^{15}N,^{13}C or both), *E. coli* BL21 DE3 were transformed with pET28a-FUS RRM-RGG2 (aa 260-390) and pET28a-FUS-RRM-RGG2-ZnF (aa 269-454) and selected on Luria Bertani agar plates in presence of 50 ug/mL Kanamycin. Expression was performed in M9 minimal media supplemented with ^{15}N ammonium chloride (1g /L) and or ^{13}C glucose (2g /L). The medium was further supplemented by traces elements and vitamin mixtures. For the expression of ^{2}H, ^{15}N, ^{13}C ILV protein, transformed E. coli strains were selected on solid medium and first grow in M9 minimal medium. Bacteria were progressively moved towards a M9 minimal medium prepared with D_20. One hour before induction of protein expression 100 mg/L of alpha-ketobutyric acid (methyl-^{13}C, 99%; 3,3-D2, 98%, Cambridge Isotope Laboratory) and 60 mg/L of alpha-ketoisovaleric acid (^{13}C5, 98%; 3-D1, 98%, Cambridge Isotope Laboratory) to ensure specific ^{13}C labelling of ILV methyl groups. Expression and protein purification were performed as already described [5].

Cell culture and plasmid transfections

HeLa, HEK 293T, and SH-SY5Y cells were cultivated in Dulbecco's modified Eagle's medium (DMEM) supplemented with 10% fetal calf serum (FCS), penicillin (100 IU/mL), and streptomycin (100 µg/mL) (DMEM$^{+/+}$) and grown at 37°C and 5% CO_2. Plasmid DNA transfections were performed with Dogtor [OZ Biosciences] and Lipofectamine 2000 [Invitrogen] according to the manufacturer's instructions. Human iPSCs [iPS-DF6-9-9T.B, WiCell] were cultured on Matrigel [Corning, 354277] coated plates in mTeSR Plus [Stemcell Technologies, 05825] and were kept under hypoxic conditions 5% O_2, 5% CO_2 and 37°C.

Lentiviral transduction

Lentiviruses were produced in HEK293FT cells grown in heat-inactivated tetracycline free DMEM supplemented with 500 µg/ml G418 to select for large T-antigen expression. 14 µg pLVX-optFUS$_{242-526}$-GSG$_{15}$-TS or pLVX-optFUS-GSG$_{15}$-TS were transfected using the Lenti-X HTX packaging mix according to the manufacturer's manual [Clonetech]. Alternatively, 20 µg pLVX-optFUS$_{242-526}$-P525L-NES-GSG$_{15}$-TS and 60 ug Packaging mix [GE, Pharmacon] were transfected to HEK293FT cells using Dogtor [OZ Biosciences] in a 150cm^2 flask. The viral supernatants of days 2 - 4 post transfection were passed through PES 0.45 µm filters and concentrated using Lenti-X concentrator following the manufacturer's instructions [Clonetech].

The parental SH-SY5Y KO A4 cell line [4] was transduced using a triple shot of concentrated virus delivered on three consecutive days. To this end, the viral pellet of each supernatant was resuspended in 3.5 ml heat inactivated DMEM supplemented with Polybrene [Sigma] and 0.5, 1.0 and 2.0 ml were transferred to the cells. Following transduction, 1.0 ng/µl Puromycin was added to the medium to select for stable integration of the lentivirus for 2 days.

***In vitro* transcription and labelling of antisense snRNAs**

To generate the probes for in situ hybridisation, we *in vitro* transcribed antisense U1, U4atac and U5 snRNA from the SP6 or T3 promoters of pcDNA3.1-U1, pUC57-U4atac and pUC57-U5-E linearized

with HindIII or BamHI, respectively. The transcription reaction was performed in 1x transcription buffer [Thermo] supplemented with 1mM rATP, 1mM rCTP, 1mM rGTP, 1mM 5-Ethynyl-UTP, 0.1 unit/µl SUPERaseIN RNase inhibitor [Ambion], 1unit/mL units inorganic pyrophosphatase [NEB] and 30 ng/µl linearized template DNA. After equilibration at 37°C for 30 minutes, 0.6 units/µl SP6 or T3 polymerase [Thermo] were added to the reaction, followed by incubation at 37°C for 45 minutes. Then, polymerase was supplemented to 1.2 units/µl and the reaction was continued for another 45 minutes. DNase treatment was performed with 0.03 units/µl Turbo DNase [Ambion] at 37°C for 30 minutes. The 5-Ethynyl-labelled RNA was then purified using the MEGAclear Transcription Clean-Up kit [Life technologies] according to the manufacturer's instructions. Subsequently, the BaseClick RNA labelling kit [BaseClick GmBH] was used for couple 6-FAM-Azide to the Ethynyl-UTPs via click reaction. Unincorporated 6-FAM-azide was removed by purification using the MEGAclear Transcription Clean-Up kit.

Preparation of cell extracts

Cell pellets were resuspended in 1 mL RIPA buffer [Thermo] (supplemented with 2x HALT protease inhibitor) / 10^7 cells, vortexed well and incubated on ice for 20 minutes. The lysates were subsequently cleared by centrifugation at 15,000 g and 4°C. Alternatively, cells were lysed in 1 mL gentle hypotonic lysis buffer (10 mM Tris, 10 mM NaCl, 2 mM EDTA, 0.5% Triton-X-100, 2x HALT protease inhibitor) / 10^7 cells and incubated on ice for 15 minutes. Then, the salt concentration was adjusted to 150 mM by addition of 5M NaCl and incubated on ice for another 5 minutes. Cellular debris was removed by centrifugation at 15,000 g and 4°C.

Immunoblotting

Unless stated otherwise, extracts were boiled in 2x NuPAGE LDS sample buffer [Life Technologies] (supplemented with 50 mM DTT) and 1 x 10^5 – 2 x 10^5 cell equivalents were separated on 4-12% NuPAGE Bis-Tris Midi Gels [Life Technologies] in MOPS buffer (50 mM MOPS, 50 mM Tris, 0.1% SDS, 1 mM EDTA, pH 7.7). For transfers, the iBlot Gel Transfer Device [Life Technologies] was used according to the manufacturer's manual. Membranes were blocked with 5 % non-fat dry milk in TBS-

T (0.1 % Tween in TBS) and subsequently incubated with the primary antibodies over night at 4°C. After washes in TBS-T, the membranes were incubated with the IRdye-labelled secondary antibodies for 1.5 hours at room temperature. The dried membranes were analysed with the Odyssey Infrared Imaging System [Li-Cor]. Antibodies are listed in Supplementary Table 2.

Immunofluorescence

Cells were seeded in 8-well chambers [Bioswisstec AG] and fixed 24 hours later with 4% PFA for 20 minutes. After three washes with TBS, the cells were permeabilized and blocked with 1x TBS, 0.5% Triton-X-100, 6% BSA for 1 hour at room temperature. Primary antibodies were diluted in $TBS^{+/+}$ (1x TBS, 0.1% Triton-X-100, 6% BSA) and incubated over night at 4°C. Unbound antibodies were removed by 3 x 5 minutes washes with $TBS^{+/+}$ at room temperature and secondary antibodies were added in $TBS^{+/+}$. After incubation at 37°C for 1.5 hours and room temperature for 30 minutes, the slides were washed with TBS 3 x 5 minutes and mounted with Vectashield HardSet mounting medium containing DAPI [Vectorlabs].

Recombinant protein expression

GB1-His-$FUS_{269-454}$-TS proteins were expressed in e.coli arctic express (DE3)RIL [Agilent]. Transformed clones were grown at 30°C in 600 mL LB medium supplemented with Kanamycin (50 µg/ml) and 1 mM $ZnSO_4$. Protein expression was induced for 3 hours by addition of 1mM IPTG at an OD_{600} of 0.6, followed by centrifugation at 4,388 x g [LYNX 6000, Sorvall] using a Fiberlite F9-6 x 1000 LEX fixed angle rotor [Thermo Scientific] for 20 minutes at 4°C. The bacterial pellets were resuspended in resuspension buffer (50 mM NaH_2PO_4, 500 mM NaCl, pH 8.0 w/ NaOH) supplemented with 1x HALT protease inhibitor [Thermo], 1 mM $MnSO_4$, 0.05 unit/µL Cyanase [Serva] and 5 mM ß-mercaptoethanol and cells were disrupted using a high pressure homogenizer [HPL6, Maximator]. To mask free Biotin, 0.2 units/µL Biolock [IBA Lifesciences] were added and incubated with the lysates head over tail for 20 minutes at 4°C, followed by centrifugation at 13,000 x g and 4°C for 15 minutes. For each construct, 1.5mL Streptactin XT High Capacity resin [IBA lifesciences] were transferred to columns and equilibrated with resuspension buffer and cleared

lysates were passed through the columns by gravity flow. After five washes with resuspension buffer supplemented with 1 mM DTT, bound proteins were eluted with resuspension buffer containing 1 mM DTT and 50 mM biotin. The pooled fractions containing the tagged proteins were subsequently passed twice through 1 mL Ni-NTA Superlow resin [IBA Lifesciences] previously equilibrated in resuspension buffer. After extensive washing with resuspension buffer, elution was performed with 50 mM $NaP0_4$ (pH 8.0), 200 mM NaCl, 300 mM imidazole, 1 mM DTT. Finally, cyanase was removed using 1 mL cyanase inactivation resin [Serva] in 3 mL resuspension buffer. To remove imidazole and exchange salt and buffering agent, the eluate was dialysed in 5 L 20 mM Hepes pH 7.3, 100 mM KCl, 1 mM DTT overnight using 10 kDa cutoff dialysis cassettes [Thermo].

FLAG-immunoprecipitation

For the FUS-FLAG immunoprecipitation experiments, 5 µg pcDNA3-EBFP-GSG15-FLAG, 10 µg pcDNA3-FUS-GSG15-FLAG and 5 µg pcDNA3-EBFP-GSG15-FLAG were transfected into HeLa FUS KO P1-A6 cells at 80% confluency in a 150 cm2 flask using Lipofectamine 2000 [Invitrogen] according to the manufacturer's instructions. All transfections were filled up to 10 µg with empty pcDNA3.1(+) and the cell culture medium was exchanged just prior to transfection. The next day, the cells were transferred into a 300 cm^2 flask and finally harvested by trypsinisation on the following day (48 hours post-transfection), washed with ice-cold PBS and shock frozen in liquid nitrogen for storage at -80 °C. To prepare protein extracts and efficiently degrade RNA, the cells were gently thawed on ice and the pellet was resuspended in 1 ml / 10^7 cells gentle hypotonic lysis buffer (10 mM Tris pH 7.2, 10 mM NaCl, 2 mM EDTA, 0.5 % Triton-X-100) supplemented with phosphatase inhibitor [Thermo Scientific], 2x HALT protease inhibitor cocktail [Thermo Scientific], 0.2 mg/ml RNase A [Sigma], 1 mM $MnSO_4$, 0.05 U/µl Cyanase nuclease [Serva]. After incubation on ice for 10 minutes, the salt concentration was adjusted to 150 mM by addition of 28 µl 5M NaCl per ml extract. Following another 5-minute incubation on ice, the extracts were distributed in 1.5 ml Eppendorf tubes and centrifuged at 4 °C and 15'000 g for 20 minutes. Then, 150 µl of the cleared lysates were removed and supplemented with 2x LDS loading buffer to serve as input fraction. To equilibrate the

matrix and remove unbound antibodies, 200 µl FLAG M2 matrix (400 µl of 50% solution) [Sigma] was washed one with 10 ml matrix preparation buffer (50 mM Tris-HCl pH 7.5, 150 mM NaCl), once with 0.2 M Glycine pH 3.5 and then twice with matrix preparation buffer. In these washing steps, the matrix was centrifuged at 4 °C and 1’000 g for 5 minutes to allow removal of the supernatant. Then, the proteins were bound to the beads by incubation on a rotating wheel overnight at 4°C. On the following day, 150 µl of the supernatants were removed and boiled with 2x LDS loading buffer to serve as post-IP fractions. The matrix was washed 5 times with HEPES NET-2 (50mM HEPES pH 7.3, 150mM NaCl, 0.1% Triton-X-100). In the last wash step, the matrix was transferred into a fresh Eppendorf tube and residual supernatant was removed in a 27-gauge syringe. Bound proteins were eluted in 200 µl HEPES NET-2 supplemented with 1 mg/ml FLAG peptide [Generon] at 4°C head-over-tail for 30 minutes. Finally, the supernatant was retrieved by passing the matrix through a Bio-Spin column [Bio Rad] at 500 g for 1 minute and boiled with 2x LDS loading buffer. All fractions were separated on 4-12% Bis-Tris gradient gels in MOPS buffer and analysed by western blotting.

Small RNA northern blot

Riboprobes were labelled by incorporation of α-32P-UTP [Hartmann Analytics] during *in-vitro* transcription. For samples, 10 *µ*g total RNA precipitate were resuspended in 20 *µ*L deionized formamide. After addition of 4 *µ*L 6× loading buffer [Fermentas], the RNA was denatured for 5 min at 95 °C and resolved on Novex TBE-Urea 6% polyacrylamide gels in 1x TBE. Then the RNA was transferred to a piece of Hybond N+ positively charged nylon membrane [Amersham Bioscience] using a TE77 ECL Semi-Dry Transfer Unit [Amersham Bioscience] in 0.5× TBE for 30 min at 3.3 mA/cm^2. The nylon membrane was dried, and the RNA crosslinked with 240 000 *µ*J UV light at 254 nm in a Stratalinker on the autocrosslink setting. For visualization, the RNA was stained with 0.03% (w/v) methylene blue in 0.3 M sodium acetate (pH 4.5). Excess staining was washed off with DEPC-treated osmotic water. The membrane was rinsed twice in 2× SSC (0.3 M sodium chloride, 30 mM sodium citrate, pH 7.0) before equilibration in prehybridisation solution (15% formamide, 10 mM sodium phosphate, in 2× SSC) for 10 min at room temperature. Then the membrane was covered with 25 mL hybridisation solution warmed to 42 °C. The radioactive riboprobes mixed in with 1 mL

hybridisation solution was then added and hybridisation was performed overnight at 42 °C under rotation. Subsequently, the membrane was washed twice for 30 min in prehybridisation solution, twice for 15 min in wash solution 1 (0.1% (v/v) Triton X-100, 2× SSC), twice for 15 min in wash solution 2 (0.1% (v/v) Triton X-100, 1× SCC), and trice for 10 min in high stringency wash solution (20% formamide, 2 mM ribonucleoside vanadyl complex, 0,05× SSC), with all the washes at 42 °C. At last the membrane was rinsed with 2× SSC, dried, and exposed to a phosphor imaging screen overnight. The signals were recorded on a BAS scanner.

Supplementary References

1 Khorshid, M., Rodak, C. & Zavolan, M. CLIPZ: a database and analysis environment for experimentally determined binding sites of RNA-binding proteins. *Nucleic Acids Res* **39**, D245-252, doi:10.1093/nar/gkq940 (2011).

2 Charenton, C., Wilkinson, M. E. & Nagai, K. Mechanism of 5' splice site transfer for human spliceosome activation. *Science* **364**, 362-367, doi:10.1126/science.aax3289 (2019).

3 Zhan, X., Yan, C., Zhang, X., Lei, J. & Shi, Y. Structures of the human pre-catalytic spliceosome and its precursor spliceosome. *Cell Res* **28**, 1129-1140, doi:10.1038/s41422-018-0094-7 (2018).

4 Reber, S. *et al.* Minor intron splicing is regulated by FUS and affected by ALS-associated FUS mutants. *EMBO J* **35**, 1504-1521, doi:10.15252/embj.201593791 (2016).

5 Loughlin, F. E. *et al.* The Solution Structure of FUS Bound to RNA Reveals a Bipartite Mode of RNA Recognition with Both Sequence and Shape Specificity. *Mol Cell* **73**, 490-504 e496, doi:10.1016/j.molcel.2018.11.012 (2019).

6 Kondo, Y., Oubridge, C., van Roon, A. M. & Nagai, K. Crystal structure of human U1 snRNP, a small nuclear ribonucleoprotein particle, reveals the mechanism of 5' splice site recognition. *Elife* **4**, doi:10.7554/eLife.04986 (2015).

7 Reber, S. *et al.* CRISPR-Trap: a clean approach for the generation of gene knockouts and gene replacements in human cells. *Mol Biol Cell* **29**, 75-83, doi:10.1091/mbc.E17-05-0288 (2018).

8 Flemr, M. & Buhler, M. Single-Step Generation of Conditional Knockout Mouse Embryonic Stem Cells. *Cell Rep* **12**, 709-716, doi:10.1016/j.celrep.2015.06.051 (2015).

9 Campagne, S. *et al.* Structural basis of a small molecule targeting RNA for a specific splicing correction. *Nat Chem Biol* **15**, 1191-1198, doi:10.1038/s41589-019-0384-5 (2019).
