# Supplementary material for: Aberrant interaction of FUS with the U1 snRNA provides a molecular mechanism of FUS induced amyotrophic lateral sclerosis
Source: Nat Commun. 2020 Dec 11;11:6341. doi: 10.1038/s41467-020-20191-3 (PMC7733473; doi:10.1038/s41467-020-20191-3)
Supplement: Supplementary file 3 — Reporting Summary [file 41467_2020_20191_MOESM3_ESM.pdf]

nature research

Corresponding author(s): Marc-David Ruepp & Frédéric Allain

Last updated by author(s): 28.10.2020

Reporting Summary

Nature Research wishes to improve the reproducibility of the work that we publish. This form provides structure for consistency and transparency in reporting. For further information on Nature Research policies, see our Editorial Policies and the Editorial Policy Checklist.

Statistics

For all statistical analyses, confirm that the following items are present in the figure legend, table legend, main text, or Methods section.

n/a	Confirmed	
☐	☒	The exact sample size (*n*) for each experimental group/condition, given as a discrete number and unit of measurement
☐	☒	A statement on whether measurements were taken from distinct samples or whether the same sample was measured repeatedly
☐	☒	The statistical test(s) used AND whether they are one- or two-sided<br>*Only common tests should be described solely by name; describe more complex techniques in the Methods section.*
☒	☐	A description of all covariates tested
☒	☐	A description of any assumptions or corrections, such as tests of normality and adjustment for multiple comparisons
☐	☒	A full description of the statistical parameters including central tendency (e.g. means) or other basic estimates (e.g. regression coefficient) AND variation (e.g. standard deviation) or associated estimates of uncertainty (e.g. confidence intervals)
☐	☒	For null hypothesis testing, the test statistic (e.g. F, t, r) with confidence intervals, effect sizes, degrees of freedom and P value noted<br>*Give P values as exact values whenever suitable.*
☒	☐	For Bayesian analysis, information on the choice of priors and Markov chain Monte Carlo settings
☒	☐	For hierarchical and complex designs, identification of the appropriate level for tests and full reporting of outcomes
☒	☐	Estimates of effect sizes (e.g. Cohen's *d*, Pearson's *r*), indicating how they were calculated

Our web collection on statistics for biologists contains articles on many of the points above.

Software and code

Policy information about availability of computer code

Data collection	All software is publicly or commercially available. See methods section for literature references. NMR data acquisition: Topspin 3.1 (Bruker). Analytical size exclusion: Unicorn (GE Healthcare). Isothermal calorimetry: Origin 7.0. Microscopy: NIS elements AR v5.01 (Nikon), LAS-X (Leica).
Data analysis	All software is publicly or commercially available. See methods section for literature references. NMR data processing and analysis: CARA v1.9, ATNOS-CANDID, CYANA 3.9, TALOS+, AMBER14. Analytical size exclusion: GraphPad CLIP-Seq and RNA-Seq: CASAVA v1.8.2 (Illumina), STAR v2.5.2a and v2.5.3a, Cutadapt 1.14, Samtools v1.8.3, Vienna RNA package v2.4.8, Meme suite v4.12.0, Snakemake v.4.3.0. Microscopy: NIS elements AR v5.01 (Nikon), LAS-X (Leica), Fiji v2.0.0.

For manuscripts utilizing custom algorithms or software that are central to the research but not yet described in published literature, software must be made available to editors and reviewers. We strongly encourage code deposition in a community repository (e.g. GitHub). See the Nature Research guidelines for submitting code & software for further information.

Data

Policy information about availability of data

All manuscripts must include a data availability statement. This statement should provide the following information, where applicable:

- Accession codes, unique identifiers, or web links for publicly available datasets
- A list of figures that have associated raw data
- A description of any restrictions on data availability

The accession number for the FUS-RRM:SL3 structure reported in this paper is PDB: 6SNJ. The accession number for FUS-RRM:SL3 chemical shifts reported in this paper is BMRB:34427. Input total RNA-Seq data and high-confidence FUS binding sites inferred from CLIP were uploaded to GEO with the accession number GSE139263.

Field-specific reporting

Please select the one below that is the best fit for your research. If you are not sure, read the appropriate sections before making your selection.

☒ Life sciences ☐ Behavioural & social sciences ☐ Ecological, evolutionary & environmental sciences

For a reference copy of the document with all sections, see nature.com/documents/nr-reporting-summary-flat.pdf

Life sciences study design

All studies must disclose on these points even when the disclosure is negative.

Sample size	No sample size calculations were performed. Quantitative experiments were performed in triplicate, which is the standard for molecular biology experiments. We consider this sufficient due to the large effect sizes observed in our experiments. RNA FISH on tissue was performed in spinal cord sections of 5 WT and 5 'FUSDelta14' knockin mice to account for variability between animals. RNA FISH and immunostainings on cell lines were performed across three cell types with up to two replicates per cell type: HeLa cells (RNA FISH, Reber et al., 2019, https://doi.org/10.1101/806158), iPSCs and motor neurons (this manuscript).
Data exclusions	No data was excluded
Replication	The results described in this manuscript were successfully reproduced and n numbers are provided in the figure legends. All experimental parameters required for reproduction by other labs are provided in the methods section.
Randomization	Randomization is not applicable to this study, as samples were not assigned to experimental groups.
Blinding	Blinding was not relevant for this study, as no subjective rating (e.g. manual counting of features) of data was performed. Quantitative measurements were performed by machines and the effects described in qualitative experiments were obvious and of large size.

Reporting for specific materials, systems and methods

We require information from authors about some types of materials, experimental systems and methods used in many studies. Here, indicate whether each material, system or method listed is relevant to your study. If you are not sure if a list item applies to your research, read the appropriate section before selecting a response.

Materials & experimental systems

n/a	Involved in the study	
☐	☒	Antibodies
☐	☒	Eukaryotic cell lines
☒	☐	Palaeontology and archaeology
☐	☒	Animals and other organisms
☒	☐	Human research participants
☒	☐	Clinical data
☒	☐	Dual use research of concern

Methods

n/a	Involved in the study	
☒	☐	ChIP-seq
☒	☐	Flow cytometry
☒	☐	MRI-based neuroimaging

Antibodies

Antibodies used	Mouse anti-FUS (4H11, Santa Cruz, sc-4711), Rabbit anti-FUS (Novus Biologicals, NB100-565), Rabbit anti-FUS (Serum 6, home-made, , described in doi: 10.1093/nar/gkv794), Mouse anti-Snurportin-1 (B-12, Santa Cruz, sc-137133), Goat anti-TIAR (C-18, Santa Cruz, sc-1749), Mouse anti-Islet-1/2 (DSHB, 39.4D5), Mouse anti-TwinStrep (IBA life sciences, 2-1507-001), Mouse anti-GAPDH (Santa Cruz, sc-32233), Mouse anti-Tub1A2 (Sigma, T9028), Rabbit anti-hnRNP H (Bethyl Laboratories, A300-511A), Rabbit anti-hnRNP A2/B1 (Abcam, ab31645), Rabbit anti-hnRNP M (Proteintech, 26897-1-AP), Rabbit anti-TAF15 (Abcam, ab133760), Mouse anti-FLAG (M2, Sigma, F1804), Mouse anti-Beta3-tubulin (2G10, Sigma, T8578), Goat anti-Mouse IRdye 680LT (LI-COR, 926-68020), Goat anti-Mouse IRdye 800CW (LI-COR, 926-32210), Goat anti-Rabbit IRdye 680LT (LI-COR, 926-68021), Goat anti-Rabbit IRdye 800CW (LI-COR, 926-32211), Donkey anti-Mouse AlexaFluor488 (Thermo Fisher, A21202), Donkey anti-Mouse AlexaFluor546 (Thermo Fisher, A10036), Donkey anti-Rabbit AlexaFluor488 (Thermo Fisher, A21206), Donkey anti-Rabbit AlexaFluor546 (Thermo Fisher, A10040), Chicken anti-Mouse AlexaFluor488 (Thermo Fisher, A21200)
Validation	We verified the specificity of all FUS antibodies by performing Western blots and immunostainings in knockout cell lines. For the Mouse anti-FUS (4H11) antibody, see Western blot and immunostainings in Figure 1c and Supplementary Figure 1c. For the Rabbit anti-FUS (serum 6) antibody, see immunostainings in Figures 5b-c and Supplementary Figures 8a-e. Rabbit anti-TAF15: Validation by manufacturer for western blot (correct size) and IF (correct localisation) (https://www.abcam.com/taf15-antibody-epr9196b-ab133760.pdf), and validated in our lab by shRNA mediated knockdown of TAF15 in HeLa cells followed by western blotting. Goat anti-TIAR: Validation by manufacturer (correct size), staining overlaps with other stress granule markers, has been used in 47 publications, https://datasheets.scbt.com/sc-1749.pdf. Mouse anti-Islet-1/2: Validated in original publication (https://doi.org/10.1016/0092-8674(94)90027-2), has been used in 51 publications. Mouse anti-GAPDH: Validated by manufacturer (correct

size), has been used in 2530 publications, https://datasheets.scbt.com/sc-32233.pdf. Mouse anti-Tub1A2: Fully characterised in original publication (Kreis et al. 1987, EMBO, https://doi.org/10.1002/j.1460-2075.1987.tb02550.x). Recognises a single band at the expected molecular weight, has been used in 95 publications. Rabbit anti-hnRNP H: We previously validated this antibody for Western blots using hnRNP H knockdown in HeLa cells (Reber et al. 2016, EMBO, https://doi.org/10.15252/embj.201593791), has been used in 21 publications. Rabbit anti-hnRNP A2/B1: Validation by manufacturer using knockout cell line, used in 12 publications, https://www.abcam.com/hnrnp-a2b1-antibody-ab31645.html. Rabbit anti-hnRNP M, Validated by manufacturer (correct size, expected cellular localisation), used in 1 publication, https://www.ptglab.com/products/HNRNPM-Antibody-26897-1-AP.htm#datasheet. Mouse anti-FLAG: Validated by us using Western blot of HeLa cells transfected with FLAG-tagged constructs (see Supplementary Figure 1b), used in 4031 publications. Mouse anti-TwinStrep: Validated by us using Western blot of SH-SY5Y cells stably expressing TwinStrep-tagged constructs (correct size) versus non-expressing control (absence of signal). Mouse anti-Snurportin-1: Validated for IF and Western blot by manufacturer (https://datasheets.scbt.com/sc-137133.pdf). Mouse anti-Beta3-tubulin: Validated in original publication (Lee, M.K., et al., Cell Motil. Cytoskeleton, 1990, doi: 10.1002/cm.970170207).

Eukaryotic cell lines

Policy information about cell lines

Cell line source(s)	HeLa (ATCC;CCL2) and 293FT (Invitrogen, Carlsbad, CA) for virus production were a gift from Oliver Muehlemann (University of Bern). SH-SY5Y FUS KO cells were described and validated in (Reber et al., 2016) and were generated from SH-SY5Y (ATCC CRL-2266). The iPSC line DF6-9-9TB was purchased from WiCell Research Institute (Madison, USA). HeLa TS-FUS and HeLa TS-FUS mutRRM mutZnF cell lines were created in our lab as described in (Humphrey et al., 2020, NAR)
Authentication	Our HeLa and SH-SY5Y cells were authenticated through STR profiling by Microsynth (Balgach, Switzerland). Our HeLa cells matched 93.8% to the DNA profile of the ATCC CCL-2 HeLa cell line. The SH-SY5Y cells matched 100% to the DNA profile of the ATCC CRL-2266 cell line. The 293FT cells used for virus production were not authenticated. HeLa TS-FUS and HeLa TS-FUS mutRRM mutZnF cell lines were validated by Western blotting and by assessing the autoregulation of endogenous FUS mRNA levels as a functional readout of exogenous FUS as described in (Humphrey et al., 2020, NAR).
Mycoplasma contamination	All cell lines tested negative for mycoplasma.
Commonly misidentified lines (See ICLAC register)	None of the cell lines used in this study are commonly misidentified

Animals and other organisms

Policy information about studies involving animals; ARRIVE guidelines recommended for reporting animal research

Laboratory animals	Spinal cord from the mouse line B6N;B6J-FUS<tm1Emcf/H>, refered to in this manuscript as FUS Delta14, were used. This line is maintained on a congenic C57BL/6J background. The tissue used in this study was generated before (Devoy et al, 2017, Brain; PMID: 29053787). The initial study cohort was aged to 18 months of age and comprising of 20 animals, of which there were 10 animals per genotype (heterozygous FUS Delta14 and wildtype littermates) with 5 males and 5 females per genotype group.
Wild animals	The study did not involve wild animals
Field-collected samples	The study did not involve samples collected from the field
Ethics oversight	All applicable international, national and institutional guidelines, including ARRIVE guidelines, for the care and use of animals were followed. Tissue was harvested according to these guidelines and according to UK home office regulations.

Note that full information on the approval of the study protocol must also be provided in the manuscript.
